# Intrauterine fetal deaths related to antiphospholipid syndrome: a descriptive study of 65 women

**DOI:** 10.1186/s13075-018-1745-2

**Published:** 2018-11-06

**Authors:** Mériem Belhocine, Laetitia Coutte, Nicolas Martin Silva, Nathalie Morel, Gaëlle Guettrot-Imbert, Romain Paule, Claire Le Jeunne, Micaela Fredi, Michel Dreyfus, Jean-Charles Piette, Odile Souchaud-Debouverie, Catherine Deneux-Tharaux, Vassilis Tsatsaris, Emmanuelle Pannier, Véronique Le Guern, Nathalie Costedoat-Chalumeau

**Affiliations:** 1 AP-HP, Cochin Hospital, Internal Medicine Department, Centre de référence maladies auto-immunes et systémiques rares d’île de France, 27 Rue du Faubourg Saint Jacques, 75014 Paris, France; 2 Caen University Hospital, Internal Medicine Department, Caen, France; 3 Spedali Civili di Brescia, Rheumatology and Clinical Immunology Department, Brescia, Italy; 4 Caen University Hospital, Gynecology and Obstetrics Department, Caen, France; 5 AP-HP, Pitié-Salpétrière University Hospital, Internal Medicine and Clinical Immunology Department, Centre de référence maladies auto-immunes et systémiques rares de l’île de France, Université Paris 6, Paris, France; 6 Poitiers University Hospital, Internal Medicine Department, Poitiers, France; 7 INSERM U1153, Obstetrical, Perinatal and Paediatric Epidemiology (EPOPé research team), DHU Risks in Pregnancy, Université Paris Descartes, Paris, France; 8 AP-HP, Cochin Hospital, Port-Royal maternity, Gynecology and Obstetrics Department, DHU Risks in Pregnancy, INSERM U1139, Paris, France; 9 INSERM U1153, Center for Epidemiology and Statistics Sorbonne Paris Cité (CRESS), Paris, France

**Keywords:** Antiphospholipid syndrome, Lupus, Thrombosis, Pregnancy, Preeclampsia, HELLP, Intrauterine fetal death

## Abstract

**Objective:**

Although one of the three obstetric manifestations of antiphospholipid syndrome (APS) is intrauterine fetal death (IUFD), little is known about it in this context. We report the first large series of patients with APS and IUFD.

**Methods:**

We retrospectively analyzed the history and clinical data of women at four French hospitals. All had (1) APS diagnosis (Sydney criteria) and (2) IUFD at or after 10 weeks of gestation (weeks) between 2000 and 2016.

**Results:**

The study included 65 women. Their median age at the index IUFD was 29 years (IQR 26–33); 38 (58%) were primigravidas. The index IUFD was the first APS clinical manifestation in 48 women (74%). Overall, 35% had a triple-positive antibody profile.

IUFD occurred at a median gestational age of 24 weeks (IQR 18–27) and was associated with maternal obstetric complications in 16 women (25%), namely, preeclampsia (*n* = 12), hemolysis, elevated liver enzymes, and low platelet syndrome (HELLP) (*n* = 6), and/or placental abruption (*n* = 5). Half of the 50 women with available data had a small-for-gestational-age fetus.

Overall, including during the follow-up period of 4 years (IQR 2–9), 28 women (43%) had at least one thrombosis, and 29% were diagnosed with systemic lupus erythematosus (SLE). Ultimately, 54 women (83%) had at least one live birth. Only one woman had three consecutive early miscarriages.

**Conclusion:**

IUFD was most often the inaugural sign of APS. Of the APS classification criteria, IUFD, preeclampsia, and thromboses were common in this cohort, while the “3 consecutive early miscarriages” criterion was met only once. With treatment, most of the women successfully had at least one live birth.

## Background

Antiphospholipid syndrome (APS) is defined by a combination of arterial and/or venous thrombosis, pregnancy morbidity, and persistent antiphospholipid antibodies (aPL), that is, lupus anticoagulant (LA), anticardiolipin antibodies (aCL), or anti-ß2 glycoprotein-1 antibodies (anti-ß2GP1), alone or in any combination [1]. According to the APS classification criteria established in 2006, the three defining pregnancy manifestations are an unexplained intrauterine fetal death (IUFD) at or after 10 weeks of gestation (weeks), a preterm birth at or before 34 weeks because of severe preeclampsia or recognized features of placental insufficiency, or at least three unexplained and consecutive pregnancy losses before 10 weeks [1]. IUFD is the obstetric complication most strongly associated with APS [2].

Although a triple-positive aPL test or positive results for LA alone put women at high risk of obstetric morbidity, including IUFD, the precise aPL antibody profiles of women with IUFD are unknown [3–7]. Moreover, available studies focus on reporting the prevalence of IUFD in APS, but no large dedicated series has described the clinical features of patients with APS and IUFD [8, 9]. Similarly, accurate data remain unavailable about the term at IUFD, the associated risks of thrombosis and of systemic lupus erythematosus (SLE), and the overall obstetric prognosis of these women with APS.

To improve our knowledge of these issues, we retrospectively analyzed fetal deaths in a large series of women with APS, and we report on their thrombotic risk, development of SLE, and their success in child-bearing.

## Patients and methods

This retrospective study took place in four internal medicine departments in France. A file review identified women who met the following inclusion criteria: (1) had an IUFD at or after 10 weeks, (2) between 2000 and 2016, and (3) fulfilled the revised classification criteria for APS [1]. Accordingly, we excluded women with an IUFD otherwise explainable by a fetal or obstetric complication unrelated to APS. We included primary APS and APS associated with SLE, defined according to the Systemic Lupus International Collaborating Clinics (SLICC) criteria [10].

As recommended by the International Society on Thrombosis and Hemostasis, the presence of LA was explored by assays of activated partial thromboplastin time (APTT) and dilute Russell’s viper venom time (dRVVT) in most patients and occasionally with the dilute prothrombin time and kaolin clotting time [11]. Both IgG and IgM isotypes were measured for aCL and anti-ß_2_GP1 antibodies. Results for aCL were considered positive when they exceeded the 99th percentile of the laboratory control values, or if the laboratory had none, when they exceeded 40 G or M phospholipids. The upper reference limits supplied by the laboratory performing the test were used for the anti-ß_2_GP1 antibodies. In accordance with the classification criteria [1], women were included only when at least two laboratory tests were positive 12 weeks apart or more and within 5 years of the qualifying event. Due to the retrospective design of the study, autoantibody testing was not centralized.

We retrospectively collected clinical, laboratory, and ultrasonography data from medical charts, treatment data during pregnancy, and placental histologic and fetal autopsy findings when available. We considered that women had been treated during pregnancy if they had been prescribed low-dose aspirin (LDA) and/or low-molecular-weight heparin (LMWH) before 12 weeks. Only Doppler data obtained at or after 22 weeks were analyzed [12].

French law does not require patients’ informed consent for medical research using data collected from retrospective chart reviews. A French ethics committee (Pitié-Salpêtrière Hospital, Paris) approved the study protocol.

### Definitions

Gestational age was estimated from the ultrasound scan performed between 11 and 14 weeks. If the first trimester ultrasound examination was not available, the date of conception was calculated based on the date of the mother’s last menstrual period. Spontaneous pregnancy loss was classified as an early miscarriage before 10 weeks and as IUFD at or after 10 weeks. Live birth before 37 weeks defined premature birth, and fetal weight estimated by ultrasound fetal biometry or birth weight below the 10th percentile for gestational age defined small-for-gestational-age (SGA) fetuses [13]. Preeclampsia was defined by hypertension (systolic blood pressure ≥ 140 mmHg or diastolic blood pressure ≥ 90 mmHg) and proteinuria ≥ 0.3 g/24 h [14].

### Statistical analyses

Categorical data are presented as percentages. Quantitative continuous data are presented as medians with their interquartile ranges (IQR). Proportions were compared between the groups using the Chi-square or Fisher’s exact test, as appropriate. *P* < 0.05 was considered statistically significant. Statistical analyses were conducted using STATA 13 software (StataCorp LP, College Station, TX, USA).

## Results

### Women’s clinical characteristics

This study included 65 women (described in Table 1). Their median age at the index IUFD was 29 years (IQR 26–33). IUFD occurred during the first pregnancy in 38 women (58%). Overall, 4 women (6%) had presented with obstetric manifestations of APS before the index IUFD, and 14 women (22%) had had a first thrombosis before the index IUFD. APS had already been diagnosed in only 11 women (17%), who had a median follow up of 6 years (IQR 3–11.5) between the diagnosis and the index IUFD, while 9 (14%) had already been diagnosed with SLE. IUFD was the first APS manifestation in 48 women (74%) and the first obstetric manifestation in 61 women (94%).

**Table 1:** Characteristics of the 65 women with an intrauterine fetal death and antiphospholipid syndrome

Age at IUFD, median [IQR]	29 [26–33]
Follow up since IUFD, median [IQR]	4 [2–9]
Gestational age at IUFD (weeks), median [IQR]	24 [18–27]
History of live birth before the IUFD, *n* (%)	11 (17)
History of live birth overall, *n* (%)	54 (83)
Antibody profile	
Anticardiolipin, *n* (%)	46 (71)
Anticardiolipin IgG	43 (66)
Anticardiolipin IgM	8 (12)
Lupus anticoagulant, *n* (%)	47 (72)
Lupus anticoagulant - aPTT-based and dRVVT assay	34 (52)
Lupus anticoagulant-dRVVT assay	13 (20)
Anti-β2GP1, *n* (%)	33 (51)
Anti-β2GP1 IgG	31 (48)
Anti-β2GP1 IgM	8 (12)
Triple-positive, *n* (%)	23 (35)
History of thrombosis before the IUFD, *n* (%)	14 (22)
History of thrombosis overall, *n* (%)	28 (43)
Associated SLE before the IUFD, *n* (%)	9 (14)
Associated SLE overall, *n* (%)	19 (29)

*IUFD* intrauterine fetal death, *IQR* interquartile range, *SLE* systemic lupus erythematosus, *aPTT* activated partial thromboplastin time, *dRVVT* dilute Russell viper venom time

LA was positive in 47 women (72%, with 13 women positive only for LA); the corresponding figures for aCL were 46 (71%, with 11 women positive only for aCL) and for anti-ß2GP1, 33 (51%, and 3 women positive only for anti-ß2GP1). In all, 23 women (35%) had triple-positive profiles and a further 15 (23%) were positive for two types of antibodies, with LA and aCL being the most frequent combination (*n* = 8). Although antibody profiles did not differ significantly according to term at IUFD (before versus at or after 18 weeks) in this population as a whole, it did differ when we excluded the women who received LDA or LMWH, treatments that might have biased the term at which IUFD occurred. In this restricted group, LA was more prevalent in the IUFD at or after 18 weeks (30% vs 72%; *P* = 0.025; Table 2) and aCL in IUFD before 18 weeks (100% vs 56%; *P* = 0.009; Table 2). Other reported APS manifestations included cardiac valve diseases (*n* = 7), thrombocytopenia (*n* = 5), livedo (*n* = 4), and chorea (*n* = 2).

**Table 2:** Antiphospholipid assays according to term at the intrauterine fetal death

	All patients^§^ (*n* = 65)			Untreated patients^a^ (*n* = 49)		
	IUFD < 18 weeks *n* = 16 (%)	IUFD ≥ 18 weeks *n* = 49 (%)	*P* value	IUFD < 18 weeks *N* = 10 (%)	IUFD ≥ 18 weeks *n* = 39 (%)	*P* value
Lupus anticoagulant	9 (56)	38 (78)	0.12	3 (30)	28 (72)	0.025
Anticardiolipin IgG	14 (88)	29 (59)	0.06	10 (100)	22 (56)	0.009
Anti-β2GP1 IgG	7(44)	24 (49)	NS	3 (30)	16 (41)	0.72
Triple- positive	6 (38)	17 (35)	NS	1 (10)	9 (23)	0.66

*IUFD* intrauterine fetal death, *weeks* weeks of gestation, *NS* not significant

^a^Patients who received no aspirin or low molecular weight heparin

### Previous pregnancies

**Fig. 1:**
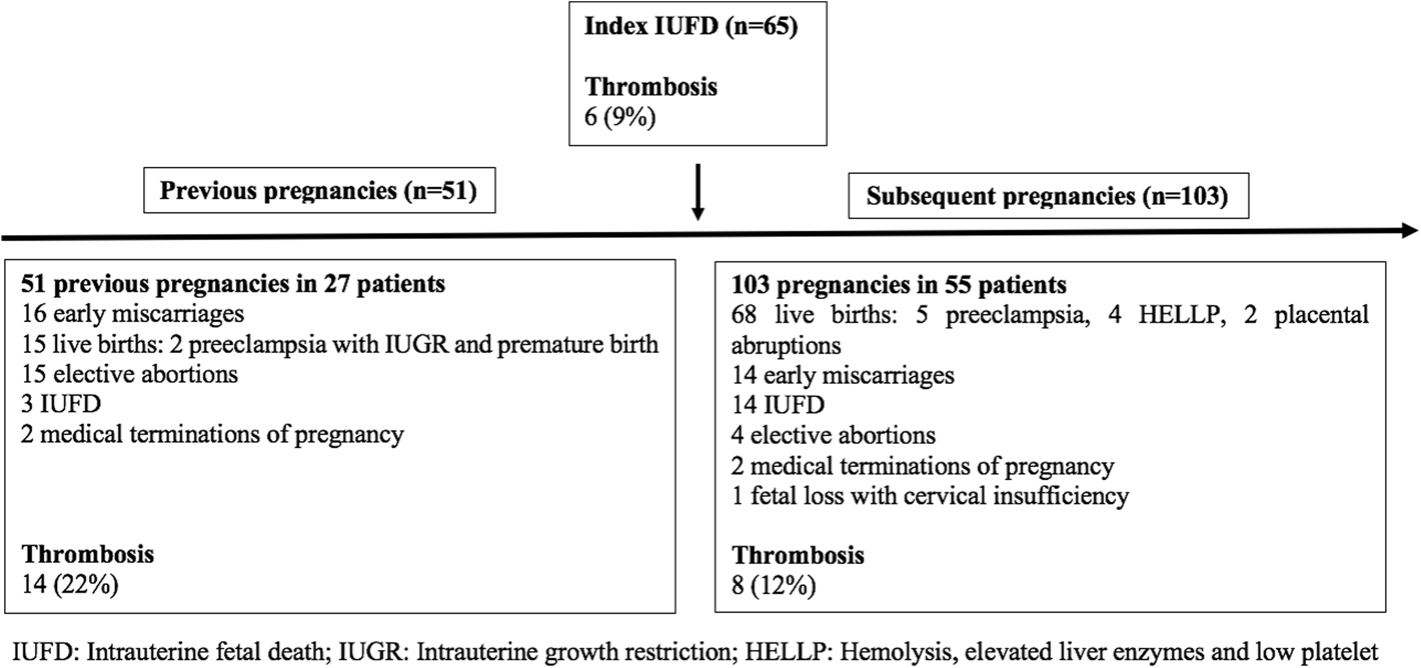
Timeline of patients’ obstetrical history

Before the index IUFD, 27 women (42%) had had a total of 51 pregnancies, including 15 live births (2 before 34 weeks, due to preeclampsia and SGA), 16 early miscarriages, 15 elective abortions, 2 medically indicated terminations of pregnancy (one for a fetal chromosomal abnormality and one for HELLP with preeclampsia), and 3 IUFD that were not index cases (one because it occurred before 2000 and 2 without any aPL test within 5 years) (Fig. 1). The 15 live births had occurred in 11 women (17%), an average of 4 years (IQR 3–6, range 1–16) before the index IUFD, and the 16 early miscarriages in 12 women.

### Description of the index pregnancy with IUFD

**Fig. 2:**
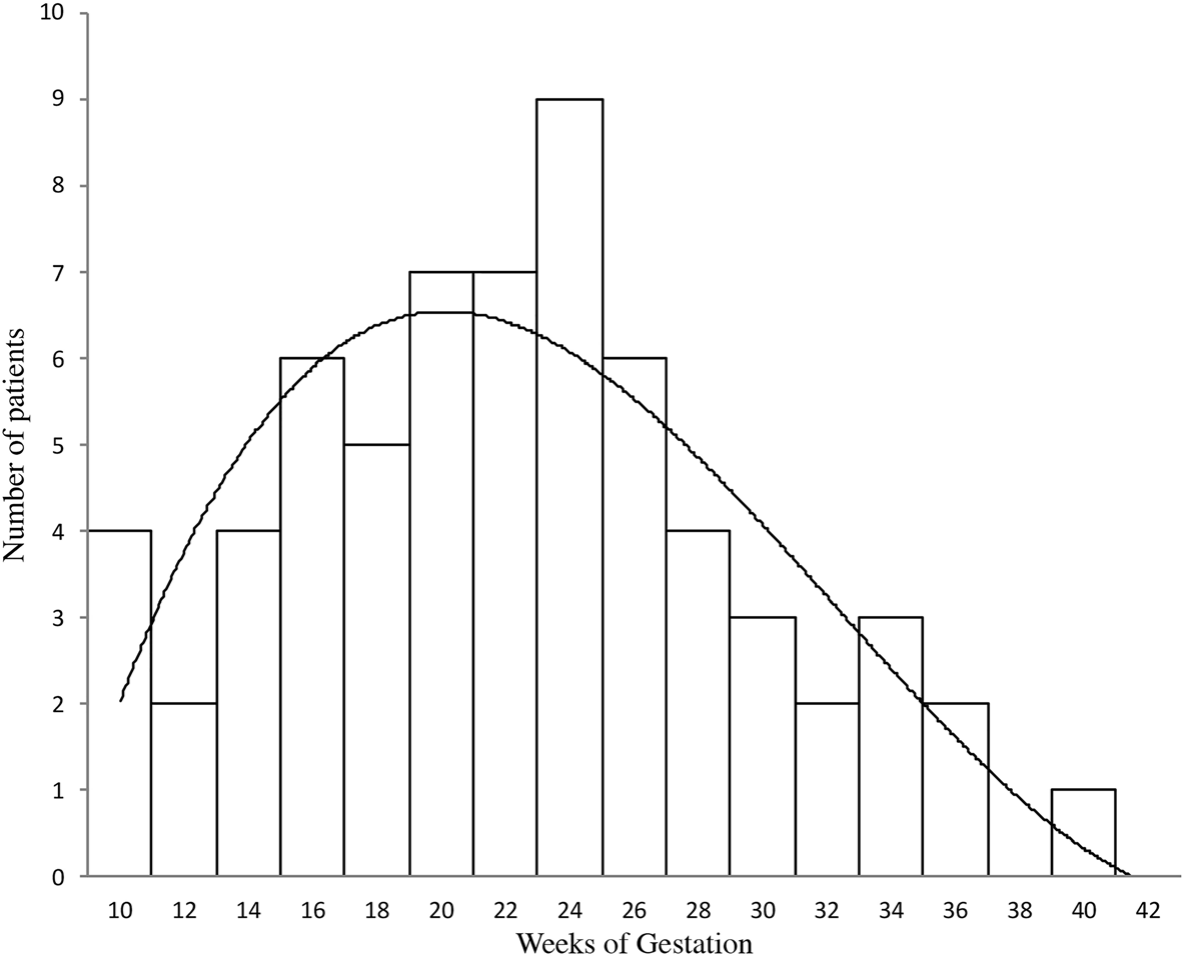
Gestational term at intrauterine fetal death

The median gestational age at loss of pregnancy was 24 weeks (IQR 18–27) (Fig. 2). Because of known APS or a history of thrombosis or miscarriage, 16 women (25%) had been treated only with LDA (*n* = 7), prophylactic LMWH (*n* = 2), therapeutic LMWH (*n* = 1), or a combination of LDA and therapeutic LMWH (*n* = 6): 3 women were also treated with hydroxychloroquine and two with corticosteroids. The other 49 women had not received treatment for this pregnancy.

Half of the 50 fetuses for whom ultrasound and/or birth weight data were available were SGA. For 18, estimated or actual birth weight was below the third percentile for gestational age. Some data were unavailable either because when APS was unknown, ultrasound examinations were only performed around 12, 22, and 32 weeks, as routinely recommended in France, or because birth weight was sometimes unavailable or not interpretable because of maceration.

Maternal obstetric complications were observed in 16 cases (25%), namely preeclampsia (*n* = 12), HELLP syndrome (*n* = 6), and/or placental abruption (*n* = 5). There was no associated maternal mortality.

Doppler ultrasound examination at or after 22 weeks was available for 10 women and was abnormal in 6 women. The abnormalities noted were bilateral uterine notches (*n* = 4) and/or reverse or absent end-diastolic umbilical flow (*n* = 3). Among these six fetuses, five (83%) were below the third percentile, and the other’s weight was normal for gestational age.

Various degrees of placental infarctions were found in 28 of the 38 cases (74%) with pathology examinations available. Other elements, such as decidual hematoma (*n* = 4), retroplacental hematoma (*n* = 3), and chronic intervillositis (*n* = 3), were also reported. In accordance with the inclusion criteria, data from the 16 available fetal pathology examinations showed no congenital malformations explaining fetal demise.

### Subsequent pregnancies

During the follow up after the index IUFD (median 4 years (IQR 2–9)), 55 women had at least one new pregnancy, with a total of 103 new pregnancies. The global outcomes were a live birth (*n* = 68, 66%), a new IUFD (*n* = 14, 14%), an early miscarriage (*n* = 14, 14%), an elective abortion (*n* = 4, 4%), a medical termination of pregnancy (*n* = 2, 2%) for SGA and placental abruption, and one fetal loss due to cervical insufficiency. Only one woman had three consecutive miscarriages. The 68 live births occurred in 51 women, and 11 involved other complications: preeclampsia (*n* = 5), HELLP syndrome (*n* = 4), SGA (*n* = 5), and/or placental abruption (*n* = 2).

**Fig. 3:**
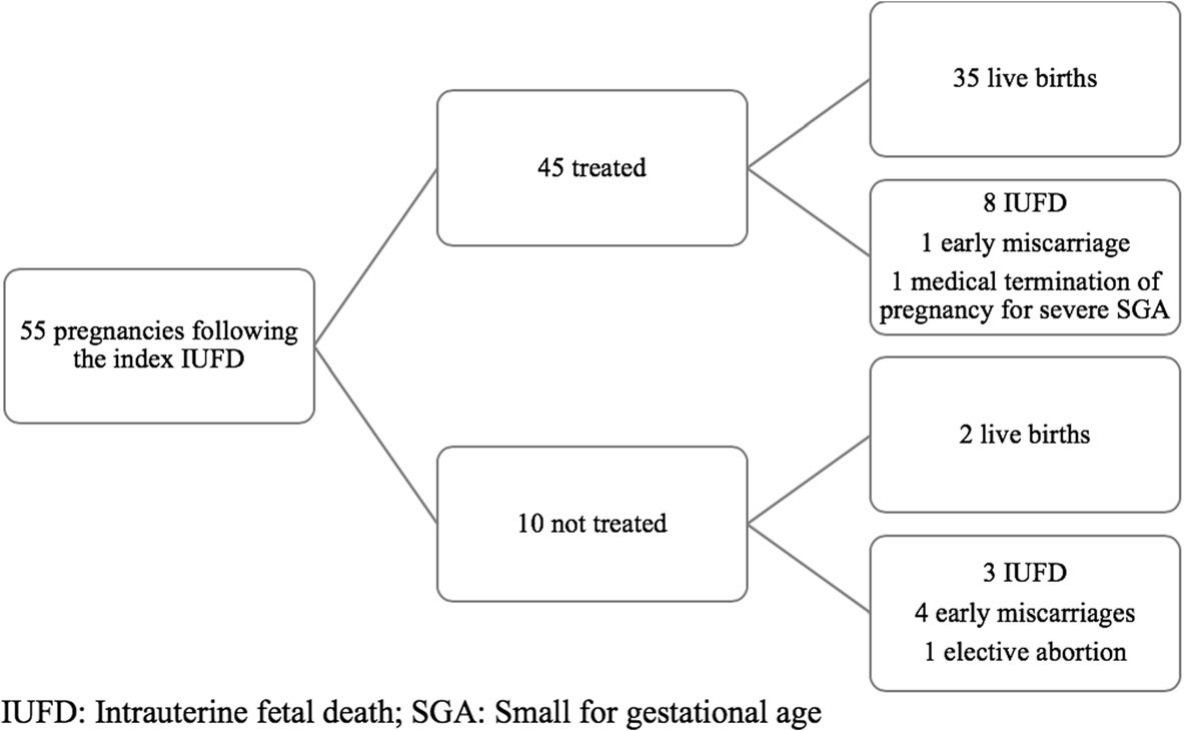
Outcome of the pregnancies immediately following the index intrauterine fetal death (IUFD)

In the first pregnancy after the index IUFD, 35 of the 45 women (78%) who received treatment (LDA and/or LMWH, started before 12 weeks) had a live birth, compared with 2 of the 10 (20%) who received neither LDA nor LMWH (because APS had not yet been diagnosed), *P* = 0.001, Fisher’s exact test (Fig. 3). Among the women who were treated, IUFD recurred in eight women. Two women had received LDA before 12 weeks, 2 others LDA and prophylactic LMWH, and 4 LDA and therapeutic LMWH. Of the other two unsuccessful pregnancies, one ended in an early miscarriage and one in a medically indicated termination of pregnancy for severe SGA.

As stated above, 11 women had had a live birth before the IUFD. Ultimately, when we considered the pre-IUFD and post-IUFD pregnancies, 54 women (83%) had at least one live birth. The median age of the 11 childless women at the last follow up was 34 years old (IQR 31–37, range 25–51). Overall, 23 women (35%) had preeclampsia, HELLP and/or a placental abruption before 34 weeks at some point in their obstetric history.

### Thrombosis

Twenty-eight women (43%) had at least one thrombotic event. The first thrombosis occurred before the pregnancy for the index IUFD (*n* = 14, 50%) or during the index pregnancy and the first 3 months postpartum (*n* = 6, 21%). Finally, the first thrombosis occurred during the follow-up period, after the postpartum period for the index IUFD, in eight women (29% of all women with a thrombosis and 12% of the entire population). Among these 28 patients, thrombosis was venous only (*n* = 17), arterial only (*n* = 6), both venous and arterial (*n* = 4), and one was a suspected microthrombosis. Among the venous thromboses, one was in the upper extremity, one cerebral, and one Budd-Chiari. The locations of arterial thrombi were cerebral (*n* = 3), cardiac (*n* = 1), mesenteric (*n* = 1), in an upper or lower extremity (*n* = 3), and multifocal (*n* = 2). Four women (6%), three of them aPL triple-positive, experienced catastrophic antiphospholipid syndrome (CAPS), all during or following a pregnancy and three associated with an IUFD.

### Associated connective tissue diseases

At the end of the follow up, 19 women (29%) had SLE, two with Sjogren syndrome as well. SLE developed before the IUFD in nine women (14%), during the index pregnancy in three (5%), and after the IUFD in seven (11%). Two other women had autoimmune hepatitis, one associated with primary biliary cirrhosis.

## Discussion

We report for the first time a large series of 65 patients with IUFD and APS with in-depth clinical context. All women met both the clinical and laboratory criteria for APS, as defined by the 2006 revised classification criteria [1].

IUFD was frequently the inaugural event of APS, its first manifestation in 48 women (74%) and its first obstetric manifestation in 61 (94%). Although 22% of the women had had a first thrombosis before the IUFD, APS was rarely diagnosed (17%) before the IUFD; in some cases, it was not diagnosed then. The presence of SLE, which mandates the research into aPL during pre-pregnancy counselling and could facilitate early diagnosis [12, 15], was reported in only nine women (14%) at the IUFD.

In all, 72% of these women had LA and 35% were triple-positive for antiphospholipid antibodies. These results are significantly higher than the 41.5% with LA in the series of 1000 patients with APS reported by Cervera et al. (27) (data on triple-positive profiles were not available in this series) (*P* < 0.001) and the 2.7% of triple-positive patients in a large cohort of 750 pregnancies in women with primary APS (*P* < 0.001) [6]. They are, however, congruent with data showing that a triple-positive profile or LA-positivity alone increases obstetric morbidity [4, 12, 16, 17] and with the finding that LA was the best indicator of poor obstetric outcome in the PROMISSE study [5, 18]. In our experience, aCL was more prevalent in IUFD before 18 weeks and LA in later fetal losses. We thus hypothesize that fetal losses before and after that term may result from different mechanisms.

Intrauterine fetal deaths occurred during the first pregnancy in 68% of our patients (elective abortions excluded) and, as stated above, was the inaugural manifestation of APS in 74%. Accordingly, during the index pregnancy, only 25% of these women received the standard treatment of LDA and/or LMWH, and only 9% received both, as currently recommended [12]. The median term at loss of pregnancy was 24 weeks (IQR 18–27). As expected, only 15 of these events happened at or after 28 weeks, most likely because fetuses with recognized signs of placental insufficiency were delivered early to avoid IUFD (and are therefore not included in this study). Half of the fetuses with available ultrasounds and/or birth weight were SGA. Because APS was frequently unknown, gaps in ultrasound monitoring could have allowed SGA fetuses to be missed (development after the last ultrasound), therefore underestimating its prevalence. Furthermore, 49 women (75%) had no preeclampsia, HELLP, or placental abruption and thus perhaps no prior warning signaling a risk of IUFD. No maternal death was observed but given that IUFD was often inaugural, any woman who died concomitantly with the IUFD most likely had not been diagnosed with APS and thus would not have been included in our study.

Doppler ultrasound data were limited, as Doppler is not routinely performed in patients without known APS. The results were abnormal in 60% of the available cases; 83% of these fetuses with abnormal Doppler findings were also severely SGA. Our sample is too small to reach any conclusions about the Doppler findings in IUFD associated with APS. We agree with current recommendations that uterine and umbilical artery Doppler scans should be performed in high-risk patients, including those with a history of thrombosis, obstetric hypertensive disorder, or fetal loss [19, 20].

Infarction and/or necrosis were reported in 28 of the 38 placentas analyzed (74%). Various histopathologic placental abnormalities have been described in patients with APS, with infarction being the most common feature, presumably due to thrombosis of the spiral arteries. This placental feature is rarely reported as occurring before 18 weeks, perhaps because the placenta villi during that period may not be vulnerable to ischemia [21]. In their meta-analysis, Viall et al. found infarction in only a third of the placentas from aPL-positive women, regardless of the pregnancy outcome, but in 71% of these placentas when associated with fetal demise. Thus, infarction seems to be the main mechanism of late fetal loss, possibly due to disruption of the annexin V shield at the syncytiotrophoblast surface [22]. Our observations are similar to those of this meta-analysis, but the size of our sample was too small to analyze the presence of infarction according to term at IUFD.

Most of the pregnancies during follow up that were treated appropriately (LDA or LMWH) were successful (78%). In Cervera’s cohort, only 47.6% of pregnancies (753/1580) at inclusion were successful, but that figure increased to 72.9% (137/188) over the next 10 years, probably reflecting the increasing effectiveness of treatment. Bats et al. found no recurrence of IUFD in a prospective follow up of 33 post-IUFD pregnancies in women with APS treated with LDA and prophylactic LMWH [23]. Overall, 83% of our patients had at least one live birth. The 11 women (17%) with no live births were mostly still of reproductive age by the end of the follow-up period (median age 34 years (IQR 31–37)). The prognosis for a pregnancy thus appears to be encouraging when appropriate treatment is implemented [24]. In light of these data and our results, we consider that women with APS who experience an IUFD should receive both LDA and LMWH for subsequent pregnancies, as well as appropriate education.

To better understand the phenotypes of women with obstetric APS, Bramham has proposed distinguishing three groups, those with (1) recurrent miscarriage, (2) late fetal loss or early delivery (< 32 weeks) due to placental dysfunction, and (3) thrombotic APS [25]. We found considerable overlap between IUFD, history of early delivery due to placental dysfunction, and thrombotic events in our patients with APS. Our additional finding of rather high frequencies of arterial thrombosis (10/65) and CAPS (4/65) underlines that women with an IUFD probably have a more severe phenotype [26], perhaps because late pregnancy losses are most likely provoked by a mechanism involving thrombosis and ischemia. By contrast, only one of our patients with IUFD had a history of three or more consecutive early miscarriages, a rate not significantly different from that observed in the general population. This finding suggests that consecutive early miscarriages and fetal deaths probably differ from both phenotypic and physiopathological points of view. Finally, because the risk of a first thrombosis after an IUFD was 12% in our series, these women should be considered at high risk and managed accordingly.

At the end of our follow up, 29% of the women had SLE; 11% had developed it during follow up, after the index IUFD. This prevalence, which does not differ from that in Cervera’s large cohort (36%; *P* = 0.26) [27], emphasizes the need for close monitoring of these women so that they can receive hydroxychloroquine to prevent SLE when appropriate [28].

Our study has some limitations. The retrospective nature of this study did not allow us to collect standardized or exhaustive data, especially on ultrasound examinations, birth weight, and placental analyses. Similarly, immunologic and hematologic assays, although performed in certified laboratories, were not centralized. Because these data were collected retrospectively, the IUFD work up was not standardized and we cannot rule out the possibility that some other women may have had other causes contributing to their IUFD. Moreover, we note that it is possible that treatment modified the course of disease in some women, as is inherent in any diagnosis, especially when, as here, the IUFD was the initial disease manifestation. Nonetheless, the strengths of our study include our patients’ moderate to strong aPL profiles confirmed on two occasions, the availability of all three aPL tests in all women, and the longitudinal obstetric histories.

## Conclusion

In summary, IUFD was most often an inaugural sign of APS and was related to maternal obstetric complications in 25% of cases. SGA fetuses represented half the cases for which data were available. The rather high prevalence of thrombosis and its occurrence at any point before, during, or after the index IUFD underlines the relationship between IUFD and thrombosis in patients with APS. Of the APS classification criteria, IUFD, preeclampsia, and thromboses were common in this cohort, while the “3 consecutive early miscarriages” criterion was met only once. Most women who received appropriate treatment successfully had at least one live birth. Nonetheless, the limited success rate for each individual pregnancy (and some women’s lack of success) and the frequency of preeclampsia emphasize the need for new therapeutic targets to improve obstetric outcome in these women.

## Abbreviations

aCL: Anticardiolipin antibodies
anti-ß2GP1: Anti-ß2 glycoprotein-1 antibodies
APL: Antiphospholipid antibodies
APS: Antiphospholipid syndrome
APTT: Activated partial thromboplastin time
dRVVT: Dilute Russell’s viper venom time
HELLP: Hemolysis, elevated liver enzymes and low platelet
IQR: Interquartile range
IUFD: Intrauterine fetal death
IUGR: Intrauterine growth restriction
LA: Lupus anticoagulant
LDA: Low-dose aspirin
LMWH: Low-molecular-weight heparin
SGA: Small for gestational age
SLE: Systemic lupus erythematosus
Weeks: Weeks of gestation
